# Synthetic Hexanucleotides as a Tool to Overcome Excessive Neutrophil Activation Caused by CpG-Containing Oligonucleotides

**DOI:** 10.3390/pathogens10050530

**Published:** 2021-04-28

**Authors:** Ekaterina A. Golenkina, Svetlana I. Galkina, Nina G. Dolinnaya, Evgenii A. Arifulin, Yulia M. Romanova, Galina F. Sud’ina

**Affiliations:** 1 Belozersky Institute of Physico-Chemical Biology, Lomonosov Moscow State University, 119991 Moscow, Russia; golyesha@mail.ru (E.A.G.); galkina@genebee.msu.ru (S.I.G.); lewanit@yandex.ru (E.A.A.); 2 Department of Chemistry, Lomonosov Moscow State University, 119991 Moscow, Russia; dolinnaya@hotmail.com; 3 Gamaleya National Research Centre of Epidemiology and Microbiology, 123098 Moscow, Russia; genes2007@yandex.ru

**Keywords:** neutrophil, modified oligonucleotides, reactive oxygen species, CpG oligonucleotides, apoptosis

## Abstract

Mimicking bacterial DNA, synthetic CpG-containing oligodeoxyribonucleotides (CpG-ODNs) have a powerful immunomodulatory potential. Their practical application is mainly associated with the production of vaccines, where they are used as adjuvants, as well as in local antimicrobial therapy. CpG-ODNs act on a wide variety of immune cells, including neutrophilic granulocytes. On the one hand, the stimulatory effect provides both the direct implementation of their antimicrobial and fungicidal mechanisms, and an avalanche-like strengthening of the immune signal due to interaction with other participants in the immune process. On the other hand, hyperactivation of neutrophilic granulocytes can have negative consequences. In particular, the formation of unreasonably high amounts of reactive oxygen species leads to tissue damages and, as a consequence, a spontaneous aggravation and prolongation of the inflammatory process. Under physiological conditions, a large number of DNA fragments are present in inflammation foci: both of microbial and self-tissue origin. We investigated effects of several short modified hexanucleotides on the main indicators of neutrophil activation, as well as their influence on the immunomodulatory activity of known synthetic CpG-ODNs. The results obtained show that short oligonucleotides partially inhibit the prooxidant effect of synthetic CpG-ODNs without significantly affecting the ability of the latter to overcome bacteria-induced pro-survival effects on neutrophilic granulocytes.

## 1. Introduction

Several decades have passed since convincing evidence was provided for the essential role of bacterial DNAs and the synthetic oligodeoxyribonucleotides (ODNs) mimicking them in the formation of the immune response. During this period, it became known that the presence of unmethylated CpG-motif is necessary for the successful activation of lymphocytes [1]. It has been shown that secondary structures formed by self-complementary palindromic regions of the CpG-ODNs are critical for their immune activity [2].

With the accumulation of experimental data, the range of clinical applications of synthetic CpG-ODNs expanded. To date, these compounds have shown high efficiency as vaccine adjuvants, in anticancer immunotherapy [3], and in the treatment of microbial and protozoal infections [4].

The Toll-like receptor 9 (TLR9) plays the most significant role in the recognition and initiation of the cellular response to bacterial DNA, host-derived denatured DNA fragments, and their synthetic analogs [5]. This receptor is widely expressed and regulates the activity of immunocompetent cells, including neutrophilic granulocytes (or polymorphonuclear leukocytes, PMNLs). The latter are pivotal not only for the first line of defense against invading pathogens; in recent years, evidence has accumulated that they are also important in the orchestration of adaptive immunity. The activation of neutrophils triggers the production of reactive oxygen species (ROS), leukotriene synthesis, and also leads to degranulation. The released metabolites ensure the recruitment and maturation of antigen-presenting cells [6]. Obviously, these cells are extremely important, both for the formation of an immune response during vaccination and for providing antimicrobial action. Since neutrophilic granulocytes are among the lymphocytes infiltrating solid tumors, regulation of their functional activity, including with the help of TLR9 ligands, enhances the therapeutic effects of anticancer immunotherapy. However, at the same time, the aberrant reactivity of neutrophilic granulocytes, especially their hyperactivation, can cause side effects of medical interventions. In particular, in excessive amounts, ROS can cause at least local tissue damage and, accordingly, prolongation of the inflammatory process [7,8]. Systemic side effects can be associated with ROS-related vascular dysfunctions.

It has been shown by Pohar J. et al. [9] that short oligonucleotides, the lengths of which vary from 2 to 6 nucleotide units, can affect the activation potential of synthetic CpG-ODNs in relation to the B-lymphocytic cell line.

In order to search for opportunities to control the stimulating potential of synthetic oligonucleotides, we investigated the joint effects of CpG-ODNs and short hexanucleotides on PMNL functions.

## 2. Results

Synthetic CpG-ODNs of A and B classes, as well as short hexanucleotides of various primary structures used in this work, are shown in Table 1.

Based on the DNA sequence, hexanucleotides with a fully phosphorothioated sugar-phosphate backbone (Table 1) can be classified into two types: xcgccc and tcxccc, where x is any purine or pyrimidine nucleoside.

We analyzed the production of reactive oxygen species, adhesiveness, and apoptosis of neutrophilic granulocytes, that is, physiological responses that are most significant for the potentiation and timely resolution of the inflammatory response.

### 2.1. Hexanucleotides Inhibit an Increase in the Production of Reactive Oxygen Species Induced by CpG-ODNs

**Figure 1:**
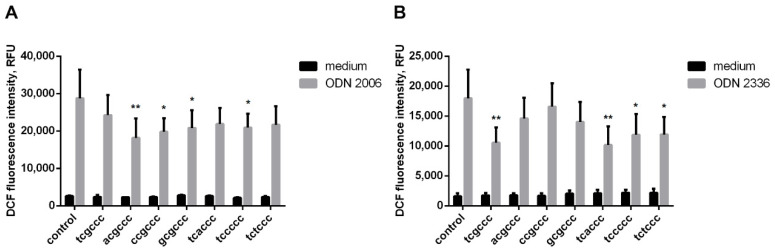
Influence of hexanucleotides on intracellular oxidant hyperproduction by neutrophilic granulocytes treated with CpG-ODNs. H_2_DCF-DA-loaded PMNLs were cultured for 1 h at 37 °C in 5% CO_2_ with (gray) or without (black) 1 µM ODN 2006 (**A**) or ODN 2336 (**B**). Short oligonucleotides were added at a concentration of 1 µM simultaneously with the CpG-ODN, except for the control (HBSS/HEPES) samples. DCF fluorescence was recorded every 10 min for an hour. Presented are average values of the increase in fluorescence intensity in relative fluorescence units (RFU) for 30 min after the indicated stimuli injection. * *p* < 0.05; ** *p* < 0.01 compared with the corresponding control, as indicated by two-way ANOVA followed by Dunnett’s multiple comparison test, n = 3 for ODN 2006, n = 5 for ODN 2336.

As previously demonstrated, the ODN 2006 is itself a potent inducer of ROS [12] (Figure 1A) and the class A ODN 2336 shows a similar effect (Figure 1B). However, the simultaneous addition of equimolar amounts of hexanucleotides listed in Table 1 leads to suppression of the prooxidant activity of both CpG-ODNs (Figure 1).

The prooxidant potential of CpG-ODNs 2006 and 2336 was found to be sensitive to the sequence of the hexanucleotides used. Short oligonucleotides of the tcxccc type inhibited ROS production induced by ODN 2336. As for ODN 2006, the hexanucleotides of another type, xcgccc, had a greater inhibitory effect.

The intracellular ROS detection method using 2′,7′-dichlorofluorescein diacetate (DCFH-DA) label is not selective; moreover, DCF is prone to photooxidation [13], but it can be used to analyze the joint production of ROS and reactive nitrogen species (Figure 1), that is, the general oxidative status of neutrophils. Taking this into account, we supplemented the study of ROS formation with an analysis based on the use of dihydroethidium (DHE). This freely cell-permeable fluorescent dye is believed to be primary specific for superoxide anion detection. For further experiments, only those hexanucleotides were selected that are active in the DCF analysis. We used two methodological approaches based on the physicochemical properties of DHE and its derivatives. DHE is known to interact with superoxide to form the ethidium cation (E^+^), which then intercalates into DNA [14]. Intercalation increases the basic fluorescence of (E^+^) at wavelengths in the range 590 to 620 nm when exposed to ultraviolet light (excitation wavelength is 500 nm). Flow cytometry with the specified parameters makes it possible to judge the intensity of intracellular superoxide ion formation. DHE also interacts with superoxide to form 2-hydroxyethidium (EOH), the yellow fluorescence of which can be visualized with excitation (480 nm) and emission (567 nm) filters. As demonstrated by Peshavarya et al. [15], this approach allows one to appreciate the extracellular release of superoxide ions in suspension cultures.

**Figure 2:**
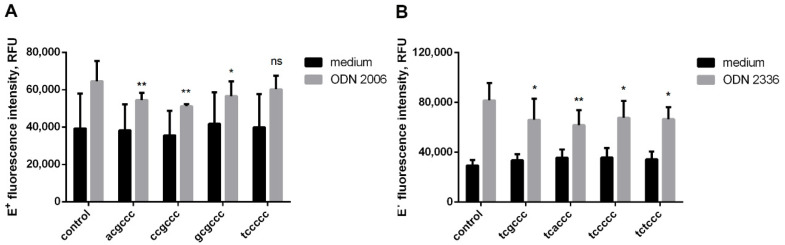
The restrictive effect of short oligonucleotides on intracellular superoxide formation under the action of CpG-ODNs. DHE-stained PMNLs (1 × 10^6^/mL) were cultured for 30 min at 37 °C in 5% CO_2_ with (gray bars) or without (black bars) 1 µM ODN 2006 (**A**) or ODN 2336 (**B**), supplemented with equimolar amounts of short ODNs as indicated, except for HBSS/HEPES control. The EOH fluorescence was then measured (ex. 488 nm, em. 610 nm) by flow cytometry. Values represent the means ± SEM of the DNA-bound ethidium fluorescence intensities, RFU. * *p* < 0.05; ** *p* < 0.01 compared with the corresponding control, as indicated by two-way ANOVA followed by Dunnett’s multiple comparison test, n = 3.

According to the results obtained, the combined action of short oligonucleotide acgccc or ccgccc with ODN 2006 markedly inhibits the intracellular production of superoxide anions, bringing it to the level of control values. Hexanucleotide gcgccc had a lesser effect, while tccccc practically did not affect the prooxidant potential of ODN 2006 (Figure 2A). ODN 2336-induced intracellular generation of superoxide anions was constrained by hexanucleotides tcgccc, tcaccc, tccccc, and tctccc, with tcaccc having the most pronounced and stable effect (Figure 2B).

**Figure 3:**
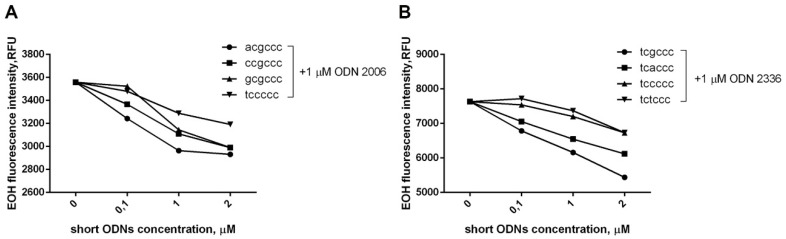
The addition of short oligonucleotide reduces CpG-ODN-induced extracellular superoxide formation in a dose-dependent manner. PMNLs suspension (1 × 10^6^ cells/mL) in DHE-containing (10 mg/mL) HBSS/HEPES buffer was dispensed into fibronectin-coated wells of a 96-well plate. The cells were cultured for 30 min at 37 °C in 5% CO_2_ in the presence of 1 µM ODN 2006 (**A**) or ODN 2336 (**B**), supplemented with various amounts of hexanucleotide as indicated. EOH fluorescence (ex. 488 nm, em. 567 nm) was recorded every 5 min for 30 min. Points on the curves correspond to the fluorescence intensity values (RFU) 30 min after the addition of stimuli. Fluorescence intensity in HBSS/HEPES control is 350 ± 47.

EOH fluorescence value correlates with the intensity of superoxide anions released into the extracellular space. It turned out that short ODNs are able to inhibit the superoxide leakage that occurs under the action of CpG-ODNs and that this ability is dose-dependent (Figure 3).

### 2.2. Hexanucleotides Reduce the Efficiency of CpG-ODN Binding to Neutrophils

**Figure 4:**
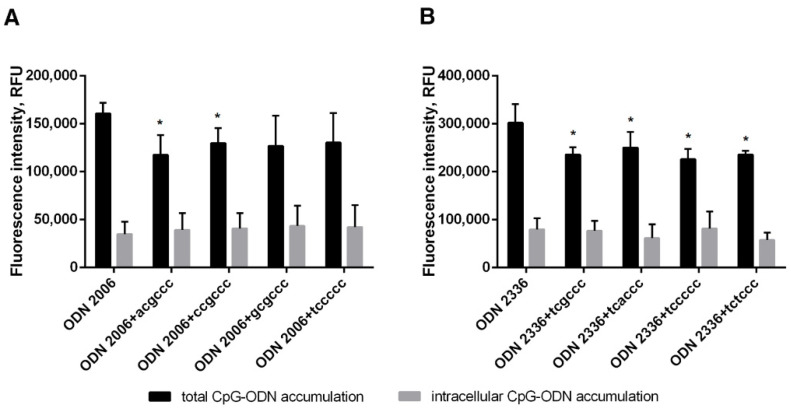
The addition of a short oligonucleotide reduces CpG-ODN surface binding to neutrophils. PMNLs suspension (1 × 10^6^ cells/mL HBSS/HEPES) was incubated for 20 min at 37 °C in 5% CO_2_ with 1 µM FAM-labeled ODN 2006 (**A**) or ODN 2336 (**B**), supplemented or not with hexanucleotide, as indicated. Then the total and intracellular oligonucleotide accumulations were analyzed by flow cytometry. The surface fluorescence was quenched with a trypan blue solution. Values represent the means ± SEM of FAM fluorescence intensities (RFU) (n = 3), * *p* < 0.05, compared to the corresponding reference value, as indicated by two-way ANOVA followed by Tukey’s multiple comparison test.

Using fluorescein-labeled ODNs 2006 and 2336, we showed that hexanucleotides reduce the amount of membrane-bound CpG-ODNs (Figure 4), which is most likely directly related to their ability to mitigate the prooxidant effects. At the same time, we did not find any suppression of CpG-ODN uptake by neutrophil cells.

### 2.3. Hexanucleotides Do Not Affect Neutrophil Adhesion Stimulated by CpG-ODNs

**Figure 5:**
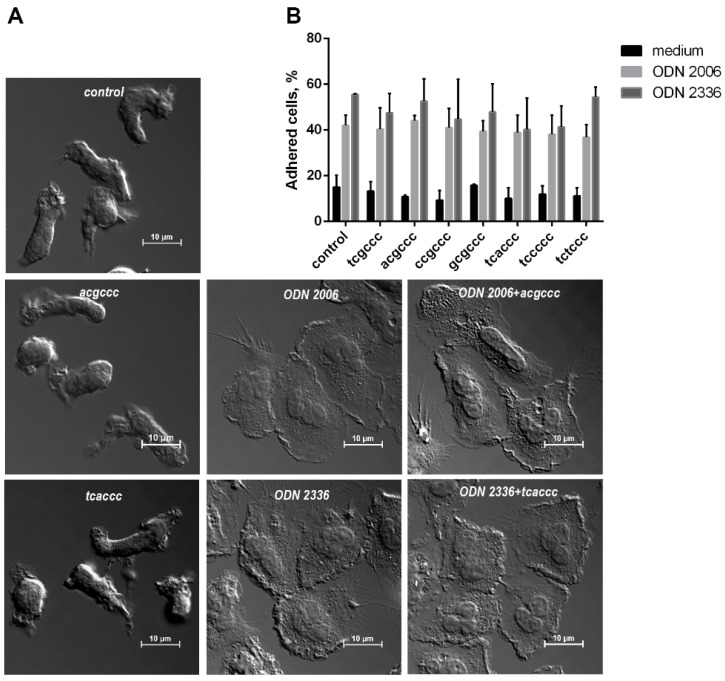
The increase in the adhesion of neutrophilic granulocytes under the influence of CpG-ODNs is insensitive to short oligonucleotides. (**A**). Differential interference contrast microscopy images of PMNLs adhered to fibrinogen-coated substrate during a 30-min incubation in the presence of ODNs or their combinations (as indicated, concentration of ODN 1 µM). (**B**). PMNLs (10^6^ cells/probe in HBSS/HEPES) were seeded in fibrinogen-coated 24-well plates and incubated for 30 min at 37 °C in 5% CO_2_ in the presence of 1 µM ODN 2006 (light gray bars) or ODN 2336 (dark gray bars) with the addition of equimolar amounts of short ODNs, as indicated, or only short ODNs (black bars), except for HBSS/HEPES control. Then, the number of adherent cells was estimated using the chromogenic assay of myeloperoxidase-coupled *ortho*-Phenylenediamine dihydrochloride oxidation. Presented are pooled data for the numbers of adhered cells, as the percentages of PMNLs initially contained in the samples, calculated according to the calibration values (n = 4).

In addition to ROS production, we also studied how the adhesiveness of neutrophilic granulocytes is altered by synthetic ODNs. In contrast to hexanucleotides, CpG-ODNs had a pronounced stimulating effect, increasing by several times the proportion of cells firmly attached to a fibrinogen-coated substrate (Figure 5). When added simultaneously in equimolar amounts, none of the short ODNs influenced the stimulating effects of ODN 2006 or ODN 2336 on the neutrophil adhesion.

### 2.4. Short ODNs Do Not Affect the Ability of CpG-ODNs to Overcome the Antiapoptotic Effect of S. typhimurium Bacteria

**Figure 6:**
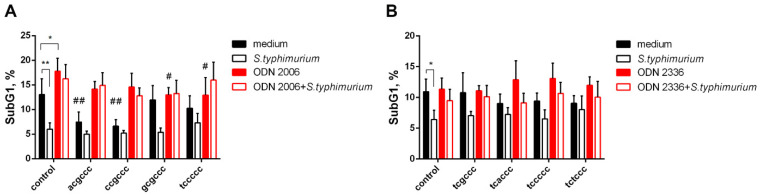
Short oligonucleotides do not interfere with the CpG-ODNs to overcome the antiapoptotic effect of *S. typhimurium*. PMNLs (10^6^ cells/probe) were incubated for 18 h at 37 °C in 5% CO_2_ in the presence of 1 µM ODN 2006 (**A**) or ODN 2336 (**B**), supplemented with equimolar amounts of short ODN, as indicated, or with only a short ODN (black bars), except for medium control. *S. typhimurium* bacteria were added at the same time as other stimuli (unfilled bars). Values represent the mean ± SEM of the apoptotic (Sub G1) neutrophil fractions (n = 4); * *p* < 0.05; ** *p* < 0.01 for pairwise comparisons; # *p* < 0.05; ## *p* < 0.01 compared to corresponding controls (two-way ANOVA, Tukey’s or Dunnett’s multiple comparison test).

An important property of CpG-ODNs, in particular ODN 2006, is their ability to stimulate apoptosis of neutrophilic granulocytes, which is caused, among other things, by the damaging effect of intracellular oxidants. With the development of an antibacterial response, the timely activation of apoptotic mechanisms contributes to the resolution of the inflammatory process. As shown by the assessment of apoptotic changes, carried out after 18 h of incubation with oligonucleotides (Figure 6), short ODNs do not affect the ability of ODN 2006 and ODN 2336 to overcome the antiapoptotic effect of *S. typhimurium* bacteria, while inhibiting prooxidant activity; the ability of CpG-ODNs to resist the survival of bacteria is retained. It also turned out that hexanucleotides acgccc and ccgccc have independent antiapoptotic effects.

To exclude the possibility that CpG-ODNs induce NETosis, thus affecting the number of viable cells, we incubated neutrophils for 5 h in the presence of oligonucleotides and (or) *S. typhimurium* bacteria, and then stained them with Hoechst 33,342 to visualize chromatin and photographed them using a fluorescence microscope. Images indicating an increase in karyopyknosis, characteristic of apoptosis [16], without elements of extracellular chromatin, characteristic of NETosis, are given in Supplementary Materials Figure S1.

## 3. Discussion

The nature and mechanisms of the immunostimulatory activity of synthetic CpG-containing oligonucleotides have been most fully investigated on cells of adaptive immunity. Neutrophilic granulocytes, whose functional responses are critical not only for innate immunity, but also for the regulation of late immune response, are sensitive targets for these ODNs that mimic bacterial DNA. Moreover, as shown by Lindau et al., neutrophils differ from other immune cells by the constitutive expression of not only endosomal, but also the surface TLR9 receptor [17].

Obviously, the foci of the immune response contain a large number of DNA fragments of both their own and pathogenic origins. Hypothetically, any of these fragments may have immunoregulatory activity. This assumption was confirmed by Pohar et al. [9], who showed an increase in the immunostimulatory effect of CpG-ODNs upon the addition of short phosphodiester oligodeoxyribonucleotides. The effect of short ODNs manifested itself in a concentrations range that was an order of magnitude higher than the concentration of stimulatory CpG-ODNs [9]. It can be assumed that such high threshold concentrations are due to the use of phosphodiester ODNs that are vulnerable to cell nucleases. In the hexanucleotides used in recent work, all phosphodiester internucleotide bonds were completely substituted by phosphorothioate ones. Their effect on the prooxidant activity of CpG-ODNs was manifested when using equimolar concentrations (1 µM) and increased in a dose-dependent manner. The CpG-ODNs used in the current study belong to two classes, differing in immunomodulatory properties and structure. ODN 2006 belongs to B-class oligonucleotides, which are unstructured and are completely phosphorothioate-modified. A-class ODN 2336 has a phosphodiester core and phosphorothioate oligoguanosine flanking regions. Due to the presence of palindromic areas, A-class ODNs are able to form stem-loop structures in contrast to unstructured B-class ODNs. These differences determine the variability in the intracellular localization of ODN interactions with TLR9 [18], the stereometry of the interactions and, accordingly, the features of ODN immunomodulatory activity. According to our results, both ODN 2006 and ODN 2336 are powerful inductors of reactive oxygen and nitrogen species in neutrophils (Figure 1, Figure 2 and Figure 3). The prooxidant activity of ODN 2006 was recorded even when it was added to neutrophilic cells at submicromolar concentrations [12]. The addition of short ODNs suppresses the excessive generation of superoxide anions, both intra- and extracellular. At the same time, an equimolar mixture of ODN 2006 and hexanucleotides containing cg steps, namely, acgccc, ccgccc, and gcgccc, suppressed the excessive intra- and extracellular production of superoxide anions. Of the short ODNs used in the study, hexanucleotides of the tcxccc type, where x is a purine nucleoside, had the most antagonistic effect on another stimulating CpG-ODN, ODN 2336. According to our results (Figure 4), hexanucleotides’ ability to inhibit the prooxidant effect is accompanied by the ability to reduce the efficiency of binding of CpG-ODN to neutrophils.

Taking into account the revealed ability to resist the prooxidant action of CpG-ODNs, we expected that short oligonucleotides would also have a noticeable effect on their proapoptotic properties, since the accumulation of oxidants often triggers apoptotic changes [19]. Indeed, by limiting the accumulation of aggressive oxidants, short oligonucleotides interfered with the proapoptotic action of ODN 2006. It is very important that hexanucleotides do not affect the ability of the CpG-ODNs to neutralize the pro-survival effect of bacteria (Figure 6).

The most essential stage in the implementation of the neutrophil functionality is the adhesion of these cells to the vascular endothelium. This allows further neutrophil migration into tissue. The ability to potentiate the adhesion of neutrophilic granulocytes is a useful property for compounds used as adjuvants. Both ODN 2006 and ODN 2336 markedly stimulate the adhesion of neutrophilic granulocytes, regardless of the presence of hexanucleotide (Figure 5).

**Scheme 1:**
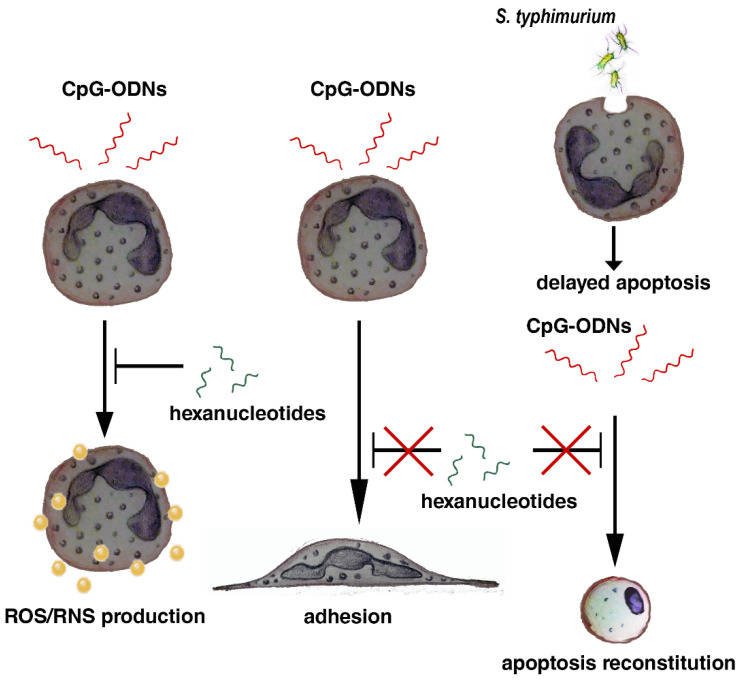
Hexanucleotides mitigate the prooxidant effect of CpG-ODNs without affecting their stimulating effect on neutrophil adhesion, and do not interfere with CpG-ODNs to overcome bacteria-mediated survival.

The results of the study are summarized in Scheme 1.

Thus, short phosphorothioate-substituted oligonucleotides can become a tool for correcting the stimulating effects of CpG-ODNs on neutrophils and help to avoid negative side effects associated with the overproduction of reactive oxygen species. However, the mechanisms of the deterrent effects of hexanucleotides on ROS overproduction are still to be determined. In addition, it is necessary to assess how CpG-ODNs affect the degranulation and microvesicle formation and whether this process can be corrected using short oligonucleotides.

## 4. Materials and Methods

The oligonucleotides including phosphorothioate-modified ones, were synthesized and purified by DNA-synthesis (Moscow, Russia).

### 4.1. Neutrophil Isolation

Human neutrophils were isolated from fresh venous blood with citrate-anticoagulant by standard techniques, as previously described [20]. Leukocyte-rich plasma was prepared by sedimentation of red blood cells with 3% T-500 dextran at room temperature. Granulocytes were then purified by low-density Ficoll-Paque (1.077 g/mL) followed by hypotonic lysis of residual erythrocytes. After washing twice in PBS, neutrophils (96–97% purity, 98–99% viability) were resuspended in Ca^2+^-free Dulbecco’s PBS containing 1 mg/mL glucose and kept at room temperature until use.

### 4.2. Intracellular ROS Detection Using 2′,7′-Dichlorofluorescein-Diacetate (DCFH-DA)

The formation of intracellular ROS was monitored by measuring green fluorescence after incorporation of carboxy-H_2_DCF-DA (Thermo Fisher Scientific, Waltham, MA, USA) according to the manufacturer’s protocol. Briefly, human neutrophils were incubated with 5 μM carboxy-H_2_DCF-DA for 60 min at room temperature, followed by a PBS wash. PMNLs were then resuspended in HBSS/HEPES, dispensed into fibrinogen-coated wells of a 96-well plate (4 × 10^5^ cells/well) and incubated at 37 °C in 5% CO_2_ in the presence of agents added according to experimental protocol. Fluorescence intensity was monitored every 10 min for one hour using a CLARIOstar microplate reader (BMG Labtech, Ortenberg, Germany) with excitation and emission wavelengths of 488 and 525 nm, respectively, for optimal monitoring of the H_2_DCF oxidation product, DCF.

### 4.3. Extracellular and Intracellular Detection of Superoxide Anion Formation Using Dihydroethidium (DHE)

#### 4.3.1. Assessment of Intracellular Superoxide Anion Production by Ethidium Fluorescence

Intracellular superoxide anion production was monitored by measuring the red fluorescence of ethidium cation intercalated in DNA, which was derived from the reaction of DHE with superoxide. PMNLs (1 × 10^6^/mL) were incubated in HBSS/HEPES supplemented with 10 µg/mL DHE and test substances. PMNLs were cultured for 1 h at 37 °C with 5% CO_2_. After stopping the reaction by cooling, the samples were analyzed on a CytoFLEX flow cytometer (Beckman Coulter, Krefeld, Germany) with excitation and emission wavelengths of 480 ± 10 and 610 ± 20 nm, respectively.

#### 4.3.2. Assessment of Extracellular Superoxide Anion Production by Hydroxyethidium (EOH) Fluorescence

PMNLs suspended in HBSS/HEPES supplemented with 10 µg/mL DHE were dispensed into fibrinogen-coated wells of 96-well plate (4 × 10^5^ cells/well) and incubated at 37 °C in 5% CO_2_ in the presence of agents added according to the experimental protocol. Fluorescence intensity was monitored every 5 min for one hour using a CLARIOstar microplate reader (BMG Labtech, Ortenberg, Germany) with excitation and emission wavelengths of 480 and 567 nm, respectively, for optimal monitoring of EOH accumulation.

### 4.4. CpG-ODNs Binding Assay

ODN 2006 and ODN 2336 labeled with 6-fluorescein amidite (FAM) at their 5′ ends were used to assess membrane binding and uptake. FAM-labeled ODNs (DNA-synthesis, Moscow, Russia) were added to cell suspension (1 × 10^6^/mL HBSS/HEPES) at a concentration of 1 µM for 30 min at 37 °C. After incubation, the samples were placed on ice, and topped with ice-cold 0.1% BSA in PBS, followed by centrifugation at 270 g. The collected PMNLs were resuspended and analyzed on a CytoFLEX flow cytometer (Beckman Coulter, Krefeld, Germany) with excitation and emission wavelengths of 480 ± 10 and 610 ± 20 nm, respectively. To distinguish between internalized and surface-bound ODNs, TB in working concentration of 1 mg/mL was used to quench surface FAM fluorescence.

### 4.5. Adhesion Assessment

#### 4.5.1. Differential Interference Contrast Microscopy of Adhered PMNLs

PMNLs (10^6^ cells/probe) were seeded on fibrinogen-precoated glass coverslips placed in culture dishes (Ø 35 mm) and incubated for 30 min at 37 °C in 5% CO_2_ in HBSS/HEPES, supplemented with ODNs according to experimental protocol. The attached cells were fixed with 2.5% glutaraldehyde in HBSS/HEPES, pH 7.3, without Ca^2+^ or Mg^2+^ ions, but containing 5 mM EDTA and 0.5 mM phenylmethylsulfonyl fluoride, metalloproteinase, and serine protease inhibitors. Permeabilized samples were analyzed on a Nikon C2 scanning confocal microscope mounted on a motorized inverted microscope (Eclipe Ti-E, Nicon, Amsterdam, The Netherlands).

#### 4.5.2. Adhesion Quantification

PMNLs (4 × 10^5^ cells/probe) in HBSS/HEPES, supplemented with ODNs according to experimental protocol, were incubated in fibrinogen-precoated 24-well plates for 30 min at 37 °C in 5% CO_2_. Supernatants were then carefully removed, and the wells were washed twice with warm PBS to remove unattached cells. Quantification of adhesion was carried out according to the method described by Ngo et al. [21]. Briefly, hydrogen peroxide (4 mM final concentration) in permeabilizing buffer (67 mM Na_2_HPO_4_, 35 mM citric acid, 0.1% Triton X-100), supplemented with 5.5 mM *ortho*-Phenylenediamine dihydrochloride (OPD), was added to substrate-bound PMNLs for 5 min. The reaction was stopped by adding of 1 M H_2_SO_4_, followed by recording the absorption values at 492 nm and comparing them with the calibration values.

### 4.6. Apoptosis Assessment

PMNL apoptosis was estimated by flow cytometric analysis of hypodiploid cells (SubG1 subpopulation) using the technique described by Nicoletti et al. [22]. Briefly, PMNLs (1 × 10^6^ cells/mL) were incubated in 24-well plates for 18 h at 37 °C in a 5% CO_2_ in RPMI-1640 medium. ODNs and *S. typhimurium* bacteria were added simultaneously at the start of incubation according to the experimental protocol. After the incubation time, the cells were harvested, supplemented with ice-cold 0.05% BSA in PBS and collected by centrifugation followed by permeabilization in cold hypotonic propidium iodide (PI) solution (20 µg/mL PI, 0.2 mg/mL RNase in 0.1% Triton X-100 in 0.1% sodium citrate). The tubes were placed at 4 °C in the dark for 10–15 min before flow cytometric analysis using a CytoFLEX flow cytometer (Beckman Coulter, Krefeld, Germany) with excitation and emission wavelengths of 480 ± 10 and 585 ± 20 nm, respectively.

### 4.7. S. typhimurium Bacteria Preparation

The *S. typhimurium* strain IE 147 was obtained from the collection of Gamaleya National Research Centre of Epidemiology and Microbiology (Moscow, Russia). Bacteria were grown in a lysogenic broth medium. Immediately before use, bacterial cells were washed twice with PBS, resuspended at a concentration of 1 × 10^9^ colony-forming units (СFU)/mL in PBS, followed by heat inactivation (90 °C, 30 min). For a more complete imitation of physiological conditions, inactivated bacteria were opsonized with 10% fresh donor serum in PBS (*v*/*v*) for 30 min at room temperature. Bacteria were then washed and resuspended in PBS.

### 4.8. Statistical Analysis

Data are expressed as mean ± SEM. Statistical data processing was performed using the GraphPad Prism software (Graph Pad Software, San Diego, CA, USA). Statistical significance for comparisons between groups was determined using two-way ANOVA followed by Tukey’s or Dunnett’s multiple comparison test. For all statistical tests, *p* < 0.05 was considered statistically significant.

## Figures and Tables

**Table 1:** Sequence and designation of oligodeoxyribonucleotides used in this study.

Sequence (5′ to 3′)	Designation
Tcgtcgttttgtcgttttgtcgtt *	ODN 2006 [10]
ggGGACGACGTCGTGgggggg **	ODN 2336 [10]
tcgccc	[11]
acgccc	[11]
ccgccc	[11]
gcgccc	[11]
tcaccc	[11]
tccccc	[11]
tctccc	[11]

* Lowercase letters denote ODN sequences with phosphorothioate backbone; ** uppercase letters denote ODN sequences with phosphodiester backbone.

## Data Availability

Data is contained within the article or supplementary material.

## Supplementary Materials

The following are available online at https://www.mdpi.com/article/10.3390/pathogens10050530/s1, Figure S1: CpG-ODN 2006 is able to overcome the antiapoptotic *S. typhimurium* effect regardless of the presence of hexanucleotide.
