# Supplementary material for: Synthetic Hexanucleotides as a Tool to Overcome Excessive Neutrophil Activation Caused by CpG-Containing Oligonucleotides
Source: Pathogens. 2021 Apr 28;10(5):530. doi: 10.3390/pathogens10050530 (PMC8146577; doi:10.3390/pathogens10050530)
Supplement: Supplementary file 1 [file pathogens-10-00530-s001.zip › pathogens-1136293-supple-revised-v1.pdf]

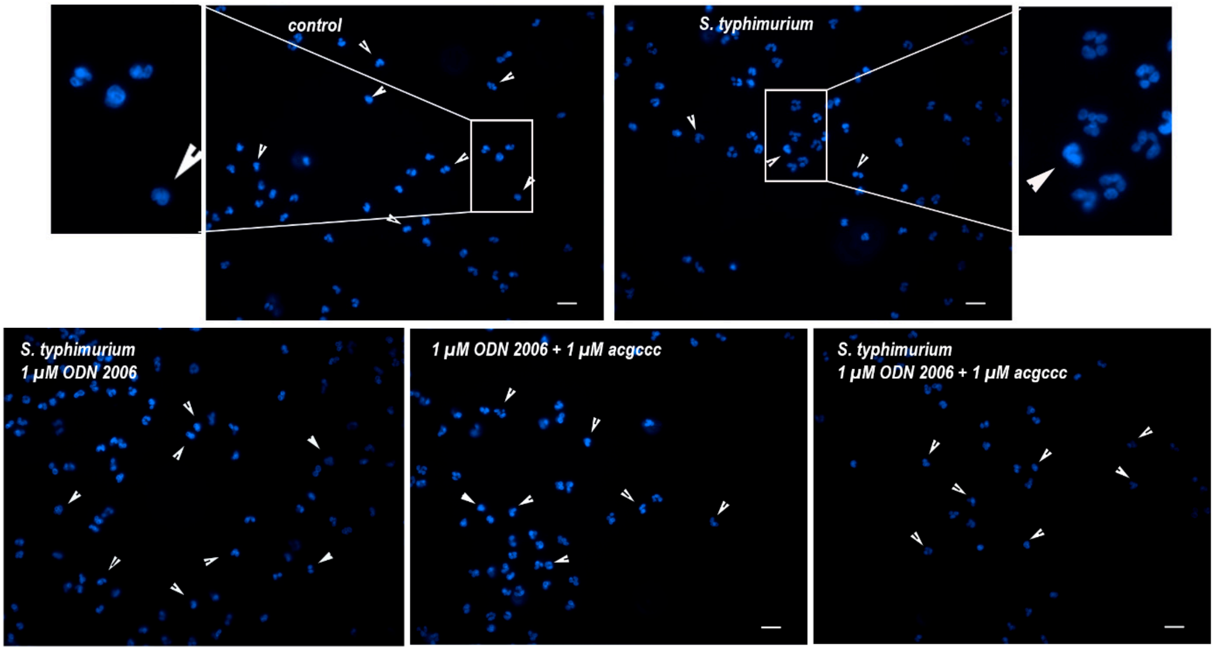

Figure S1. CpG-ODN 2006 is able to overcome the antiapoptotic *S. typhimurium* effect regardless of the presence of hexanucleotide. PMNLs (5×10^5 cells/mL RPMI 1640, 10% fetal bovine serum) were incubated in glass bottom dishes for 5 hours at 37 °C in 5% CO_2 in the presence of ODN 2006, supplemented or not with an equimolar amount of hexanucleotide (acgccc). *S. typhimurium* bacteria (at 1:10 cells per bacteria ratio) were added simultaneously with the stimuli. Unfixed cells were then stained with 0.5 µg/mL Hoechst 33342 for 10 min. Chromatin fluorescence was immediately visualized using a Zeiss Axiovert 200 M Inverted Microscope. Arrowheads indicate pyknotic nuclei. Scale bars: 20 µm. Magnification: 40×.
